# Supplementary material for: Chronic High-Dose Neonicotinoid Exposure Decreases Overwinter Survival of Apis mellifera L
Source: Insects. 2019 Dec 31;11(1):30. doi: 10.3390/insects11010030 (PMC7022569; doi:10.3390/insects11010030)
Supplement: Supplementary file 1 [file insects-11-00030-s001.zip › TableS1_TableS2.docx]

**Table S1.** Survival analysis of winter *Apis mellifera* workers chronically exposed to thiamethoxam (THI) or clothianidin (CLO) through syrup in laboratory cage trial C1.

|  | Median |  |  |  | 95% Confidence Interval | |
| --- | --- | --- | --- | --- | --- | --- |
| Treatment (ng/g) | survival time (days) | Time ratio^†^ | Standard error | P | lower | upper |
| Negative control | 16.48 | - | - | - | - | - |
| CLO 100 | 3.74 | 0.227 | 0.041 | <0.001 | 0.159 | 0.324 |
| CLO 20 | 10.27 | 0.623 | 0.077 | <0.001 | 0.489 | 0.794 |
| CLO 5 | 13.11 | 0.796 | 0.059 | 0.002 | 0.688 | 0.920 |
| THI 100 | 5.48 | 0.332 | 0.045 | <0.001 | 0.255 | 0.433 |
| THI 20 | 10.22 | 0.620 | 0.081 | <0.001 | 0.480 | 0.802 |
| THI 5 | 13.73 | 0.833 | 0.066 | 0.021 | 0.714 | 0.973 |

†The time ratio indicates the change in expected survival time for each treatment relative to the negative control by a Weibull accelerated failure time model.

**Table S2.** Survival analysis of winter *Apis mellifera* workers chronically exposed to thiamethoxam (THI) or clothianidin (CLO) through syrup in laboratory cage trial C2.

|  | Median |  |  |  | 95% Confidence Interval | |
| --- | --- | --- | --- | --- | --- | --- |
| Treatment (ng/g) | survival time (days) | Time ratio^†^ | Standard error | P | lower | upper |
| Negative control | 23.89 | - | - | - | - | - |
| CLO 100 | 5.53 | 0.231 | 0.037 | <0.001 | 0.169 | 0.317 |
| CLO 20 | 15.75 | 0.660 | 0.097 | 0.005 | 0.494 | 0.881 |
| CLO 10 | 18.92 | 0.792 | 0.036 | <0.001 | 0.724 | 0.866 |
| CLO 5 | 20.57 | 0.861 | 0.043 | 0.003 | 0.781 | 0.950 |
| THI 100 | 5.53 | 0.232 | 0.047 | <0.001 | 0.156 | 0.343 |
| THI 20 | 16.10 | 0.674 | 0.054 | <0.001 | 0.576 | 0.789 |
| THI 10 | 19.80 | 0.829 | 0.041 | <0.001 | 0.752 | 0.914 |
| THI 5 | 17.39 | 0.728 | 0.040 | <0.001 | 0.653 | 0.812 |

†The time ratio indicates the change in expected survival time for each treatment relative to the negative control by a Weibull accelerated failure time model.

|  |  |
| --- | --- |
